# Long-Term Spatial Restriction Generates Deferred Limited Space Use in a Zoo-Housed Chimpanzee Group

**DOI:** 10.3390/ani12172207

**Published:** 2022-08-27

**Authors:** Luke Mangaliso Duncan, Chiara D’Egidio Kotze, Neville Pillay

**Affiliations:** School of Animal, Plant and Environmental Sciences, University of the Witwatersrand, Johannesburg 2000, South Africa

**Keywords:** chimpanzee, primate, space use, zoo, captivity, learned helplessness

## Abstract

**Simple Summary:**

A worldwide trend amongst zoos is to replacesmall, barren enclosures with large, naturalistic ones intended to provide animals with environments which cater to their behavioural and psychological needs. Evidence suggests that naturalistic enclosures are effective but most studies focus on welfare-related behaviour or human perceptions of the enclosures. To date, little attention has been given to how animals use space in naturalistic enclosures. Our study investigated how a group of chimpanzees at the Johannesburg Zoo used space in a naturalistic enclosure by recording behaviour and space use every 5 min for an hour at a time. We found that the chimpanzees showed a preference for locations within the enclosure which coincided with their previous housing and that the chimpanzees form subgroups which conform to the space of their previous housing (i.e., small, barren enclosure). We suggest that the chimpanzees’ perception of space has been altered by their experience of the previous, smaller barren housing and that this limits their space use in the naturalistic enclosure through what appears to be a self-imposed ‘invisible cage’ around subgroups. Exactly how the ‘invisible cage’ works is unclear but our findings have implications for animal welfare, husbandry and broader conservation of endangered species.

**Abstract:**

Background: Appropriate space is considered paramount for good captive animal welfare. There has been a concerted effort by captive institutions, particularly zoos, to provide captive animals with relatively large, naturalistic enclosures which havehad demonstrated welfare benefits for animals. However, post-occupancy assessments of these enclosures tend to focus on short-term welfare-centredbehavioural effects or human perceptions of the enclosures and their effects and seldom consider spaceuse. We examined the space use of a group of eight captive chimpanzees 5 years after large-scale enclosure modification at the Johannesburg Zoo, South Africa. Methods: Instantaneous scan sampling was used to record behaviour and location of each chimpanzee at 5 min intervals in the new enclosure. From these 6.8 h of data, space-use patterns and subgroup (two or more chimpanzees within 10 m of each other) spacing were considered relative to local environmental variables, social conditions and the location and size of the previous smaller enclosures in which they had been kept. Results: Space use was heterogeneous, with some enclosure zones being used more than others, and 97.5% of subgroups restricted their spacing to the dimensions of the previous housing (10 m × 10 m). Conclusions: This pattern was not explained by individual behaviour, time of day, location, available space, weather, temperature or shade availability, inter-individual spacing or subgroup composition. We suggest the learned helplessness phenomenon may explain these observations and discuss the implications for both animal welfare and endangered species conservation.Regardless of the mechanism, we suggest that such effects could be avoided through the provision of large enclosures for captive animals.

## 1. Introduction

Spatial restriction is the hallmark of captivity. Most animals in captivity are housed in spaces that are orders of magnitude smaller than the home ranges of their free-living conspecifics [1]. Shettel-Neuber [2] describes zoo enclosure designs as falling into three distinct stages of design, based on the ideas put forward by Campbell [3]: first-generation enclosures (characterised by barred cages or steep-walled pits), second-generation enclosures (cement enclosures surrounded by wet or dry moats) and third-generation, or naturalistic, enclosures. Naturalistic enclosures have proliferated in zoos [4] and have a reputation as the most effective housing systems for zoo animals [5] as well as the most popular amongst zoo visitors [6]. Within the bounds set by practical limitations such as available space and financial costs [7], these designs seek to recreate both aesthetic and functional aspects of the natural environment [8,9] and the beneficial impacts of such enclosures are well documented across a wide range of species from amphibians [10] to apes [11]. Through the provision of appropriate behavioural opportunities mirroring the native environment of the species [12], naturalistic environments promote species-typical behaviour [13,14,15], increase overall activity and novel behaviour [16,17] and reduce abnormal [18] and potentially undesirable behaviour, such as aggression(while aggression, a natural behaviour, is not inherently problematic, it may be viewed as undesirable in captivity given the welfare risks associated with fighting and injury, as well as the potential for damage to infrastructure and harm to caregivers [8]). Given the evidence in their favour, naturalistic enclosures are becoming a standard housing system for zoo animals.

The provision of an appropriate housing space is essential for good animal welfare [19], particularly for non-human primates (hereafter ‘primates’) [20], and over time, many zoos have replaced traditional first- and second-generation enclosures with naturalistic designs. However, additional space alone is unlikely to have a meaningful impact on captive animals unless that space is used appropriately by the occupants [7]. There is a need to rigorously examine enclosure design and efficacy [21] and post-occupancy evaluations are often used to assess how appropriate new enclosures are for captive animals by identifying which aspects of enclosure designs are suitable or functional and those which may require improvement [22]. Most post-occupancy studies have focused on the behavioural impact of enclosure change, specifically on abnormal or pathological behaviour (e.g., [8]), on the perceptions of caregivers or the public of the design (e.g., [23]) or the functionality of the designs for caregivers [21] and the consequent animal welfare impact thereof [6].

By contrast, the relative impact of large-scale enclosure changes on the space use of the animals has received far less attention. It stands to reason that the substantial qualitative and quantitative modification of the physical environment involved when relatively small ‘barren’ enclosures are upgraded to large naturalistic designs renders direct comparison of the space use of subjects in the old and new environments difficult unless the original enclosure is maintained and supplemented with novel spaces and features. The type of enclosure in which an animal is housed will determine the space use of the animal [24] but there is a prevailing assumption that animals will use enclosure spaces homogenously [25]. As a general rule, heterogeneity in the physical environment acts as a determining factor of movement patterns and space use in animals [26] and space use varies both spatially and temporally according to an animal’s physiological and behavioural needs [1]. Free-ranging animal movements are affected by the distribution and encounter rate of key resources, such as food or social partners, and the quantity and quality of shelter [27] as well as factors such as relative predation [28], parasitism and disease transmission risk [29,30], energetic considerations [31] and territoriality [32]; these factors play a lesser role in determining space use in captivity, given that captive environments typically provide an adequate diet, a relatively static social and physically limited environment, an absence of predators and limited opportunities for disease and parasite transmission. Moreover, captive animal space use is affected by the size, structure and complexity of their physical housing and social context [33] and factors such as the overt presence of humans [34,35] also influence captive animal space use.

Post-occupancy evaluations to date have considered various measures of space use in new enclosures based on dividing enclosures into zones and recording the relative frequency of occupation by animals (reviewed by [36]). Based on these data, the spread of participation indices (relative use of predefined zones in an enclosure) or electivity indices (frequency of use of specific features or areas of an enclosure) can be calculated as measures of space use, or zone-use patterns can be related to behavioural expression of animals to qualify space usage [36]. Ross and colleagues applied these measures of space use to two captive ape groups, chimpanzees *Pan troglodytes* and lowland gorillas *Gorilla gorillagorilla*, through a period of a change of enclosure. Both ape groups showed distinct preferences for specific vertical tiers as well as particular physical features of their respective enclosures, including doorways and enclosure corners [37]. Ross et al. [38] compared these enclosure feature preferences in the same ape groups between the two housing conditions using electivity indices and found enclosure-specific preferences in both groups. Despite demonstrable long-term beneficial behavioural outcomes for both ape groups [11], a later quantitative study on the same subjects found the chimpanzees and gorillas used only 3.2% and 1.5% of the available space in an indoor-outdoor enclosure [39]. Studies such as these provide valuable insight into the space-use preferences of captive animals.

While the spread of participation and electivity indices provide broad measures of space use patterns and preferences for environmental features, for social species there is a further consideration which impacts space use patterns—the presence and proximity of other individuals within the group. Inter-individual spacing is governed by the distances between individuals in relation to fields of personal space and may depend on the familiarity, relative social rank, age and sex of the individuals involved, season, spatial location and the behaviour being expressed by individuals [40]. Social behaviours such as allogrooming and aggression regulate inter-individual spacing patterns [40] and are of particular importance in the context of primates and crowding in captivity [41,42,43]. In the context of enclosure use, it may be that individuals are drawn to a location because other individuals are present (i.e., through local enhancement) rather than due to a location preference per se or may have a high preference for a specific environmental feature or location but may be precluded from access due to limits on inter-individual spacing or visual access to other individuals [44]. Indeed, lowland gorillas use areas within an enclosure based on proximity to familiar individuals and avoidance of dominant individuals [45]. Thus post-occupancy evaluations should consider the role of social spacing and subgroup composition in evaluating space-use patterns for social species in order to generate a more holistic understanding of the effects of enclosure change.

With the above in mind, we investigated the space use of a group of zoo-housed chimpanzees introduced into a large naturalistic enclosure by examining both broad patterns of space use and group spacing. We quantify the daily spatio-temporal patterns of space use of the chimpanzees through zonation and a modified spread of participation index, relating these to local environmental features and the previous housing, and examine the formation and spacing of subgroups within the enclosure, relating these patterns to behaviour, local environmental variables and subgroup composition. Novelty effects are likely to impact on behaviour and space use [11,46] but the space use patterns of the chimpanzees in this study were examined 5 years following the transfer into a new enclosure and compared to the spatial characteristics of the previous small, barren enclosure. Thus, this study represents one of a handful of studies to describe long-term effects of enclosure change on space use and the first to include effects on social spacing patterns.

## 2. Materials and Methods

### 2.1. Study Subjects, Housing and Husbandry

**Figure 1:**
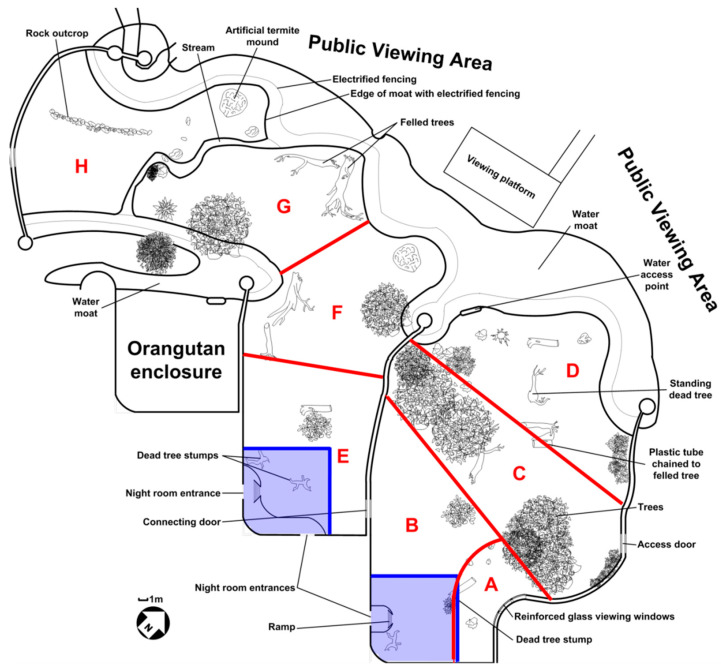
Outdoor housing area of the chimpanzee exhibit at the Johannesburg Zoo Ape House in 2009 drawn to scale. Blue lines and shading denote the location and size of the original housing areas. Red lines and large red letters denote zonation into eight unequally sized zones based on environmental elements, generalized patterns of shade availability, access to water and proximity to zoo visitors.

The study subjects were a stable group of chimpanzees, comprising four males (Thabu: 26 years; Yoda: 17 years; Amber: 10 years; Charles: 2 years) and four females (Daisy: 25 years; Zoe: 14 years; Lilly: 12 years; Joyce: 6 years). All the individuals in the group were the offspring of Thabu and Daisy and the group had been housed at the Johannesburg Zoo Ape House (26°10′06″ S, 28°01′35″ E) since birth, with the exception of one female, Lilly, who was originally part of a group of rescued orphan chimpanzees from central Africa housed at the Johannesburg Zoo [41]. Lilly was introduced into the existing family group in 2005. Given that this study was conducted in 2009, the group had been together in excess of four years and exhibited no notable social instability. Both groups were housed in a pair of identical 100 m^2^ enclosures with adjoining indoor night rooms prior to the change of enclosure in 2004; these enclosures were reconstructed and replaced by a large outdoor enclosure with a total area of approximately 2500 m^2^ [41]. The new enclosure was constructed directly over the location of the previous enclosures and retained the major structural features (i.e., barrier walls, doorways) of the previous housing (Figure 1) but was otherwise completely altered.Following the construction of the new enclosure, the group of rescued orphan chimpanzees (excluding Lilly, who was incorporated into the focal group as described above) were relocated to Chimp Eden, a sanctuary affiliated with the Jane Goodall Institute, located outside of Barberton, Mpumalanga, South Africa and thus were not subjects of this study.

The outdoor enclosure comprised two sections, approximately 1000 m^2^ and 1500 m^2^, respectively, divided by a wall with a connecting doorway and surrounded by 8 m high walls, capped with electrified fencing, or 4–5 m wide moats with 1 m high electrified fencing along the edge of the moat and 30 cm high electrified fencing extending out of the water approximately 2 m from the inner moat edge. Throughout the study, the chimpanzees had full access to both sections of the enclosure when they were outdoors. Several large living and felled trees(all of which, apart from those in zones B and E which were too small to climb, were fully accessible to the chimpanzees in their outdoor enclosures), other vegetation, many large rocks and logs were located in both outdoor sections. The smaller enclosure had a three-paneled reinforced glass viewing window, which allowed visual access for both chimpanzees and visitors alike, opposite the night room entrances as well as a large plastic barrel and tube chained to trees. The larger enclosure had a pair of artificial termite mounds. However, throughout the two years preceding this study and during this study, neither of the mounds were baited with food as enrichment.Both outdoor enclosures offered free access to water at all times. Access to two indoor night rooms was provided through passages located in the rear walls of the outdoor enclosures which corresponded to the doorways in zone B and zone E.

The feeding and husbandry regimes of the chimpanzees remained constant throughout the study. The chimpanzees were fed an assortment of foods twice daily, with morning and evening feeds comprising a similar assortment of foods which varied from day to day. Their morning feed was scattered randomly throughout their outdoor enclosures to encourage them to leave the night rooms and use the full available outdoor space. The chimpanzees had access to their outdoor enclosure between 10 h00 and 15 h00 (16 h00 on weekends), during which time keepers and animal attendants cleaned the night rooms (typically from 10 h00 to 11 h00). Similarly, their afternoon feed was spread throughout the night rooms to encourage the chimpanzees to return indoors for the night. Thus the chimpanzees were necessarily limited to either outdoor or indoor access at any given time.

### 2.2. Sampling Technique

Observation sessions were carried out on 47 randomly selected days from March 2009 throughJuly 2009. Observations were conducted between 10 h00 and 16 h00 in the outdoor enclosure only and for days where the chimpanzees only had access to the outdoor enclosures. During each sampling session, the behaviour of the chimpanzees was sampled for 60 min, using an instantaneous scan behaviour and spatial sampling technique (modified after [47]). Samples were taken every five minutes and behaviour and space-use data were recorded simultaneously. The resulting data consisted of 12 behavioural and 12 spatial records per observation session.All behavioural data were recorded according to the categories described in Table 1.Observation sessions were classified into three time categories: morning (10 h00–11 h59), midday (12 h00–13 h59) and afternoon (14 h00–16 h00).

#### 2.2.1. Spatial Sampling

The location of the chimpanzees in the enclosure was recorded at each 5 minbehavioural sampling time point. The relative positions of the chimpanzees were plotted on a scale map of the enclosure. Due to inaccuracies in the plotting of environmental features on the maps, the distances between fixed landmarks in the enclosure were measured directly in the enclosure once data collection was completed and these measurements were used to correct recorded chimpanzee positional information. Only corrected points plotted on the maps were used in later analyses. During the mapping, photographs of the chimpanzees were taken, using a Kodak C613 set at 3× optical zoom, which were used to confirm individual identities and to help ground-truth the mapping technique.

The mapping sampling technique was used to assess chimpanzee subgroups, defined as any congregation of two or more chimpanzees, each within 10 m of another chimpanzee. The influence of visual barriers wasconsidered when deciding on the limits of subgroups and possible interactions with the public, based on the findings of Bettingeret al. (visual separation was found to be important in managing aggression and spacing in captive female chimpanzees [44]). Thus, individuals outside of visual range of one another were not considered to be part of the same subgroup, regardless of the relative distances between the individuals concerned.

Excursions of individuals from subgroups were also recorded as any movement of an individual from and returning to the same subgroup that resulted in the individual exceeding a distance of 10 m from another subgroup member for no more than five minutes. If the excursion exceeded five minutes (the time to the next sampling point), the individual was no longer considered to be part of its original subgroup. However, excursions occurred rarely (<3% of all observations) and were not considered for further analysis.

#### 2.2.2. Enclosure Space-Use Patterns

Plowman [50] suggests that in order to meaningfully assess space use in captivity, division of the physical space into zones must be done with salient environmental features in mind, rather than using arbitrary equal-sized zones, a practice used in studies of animal space use in zoos (e.g., [51]). Thus, to assess the enclosure space-use patterns, the enclosure was divided into eight unequally sized zones based on environmental features (e.g., the presence of living or felled trees or rocks), gross patterns of shade distribution, access to water and relative distances to the public. The zonation is shown in Figure 1. Only for time slots where the locations of all eight individuals, regardless of whether they were in a subgroup or moving independently, were marked on the map or could be determined by examining the photographs, were zone-use patterns recorded. If two or more consecutive time slots within an observation session provided locations for all eight individuals, every second time slot was discarded, to minimize interdependence between time slots.

The resulting dataset of 82 time slots (approximately 6.8 h of data) was then used to calculate the spread of participation index (SPI) using a modified equation for unequal zone size (as described by [50]). The index provides an estimate of space-use bias by generating a value between 0 and 1, with 0 indicative of no space-use bias (all zones used equally) and 1 suggesting extreme bias (all observations in one zone). The SPI value for the chimpanzee group data was 0.43, suggesting a moderate degree of space-use bias, and so space-use was interrogated further (see Data analysis).

#### 2.2.3. Subgroup Spacing Patterns

Very few, and inconsistent, data exist regarding subgroup spacing patterns for chimpanzees in nature [52,53] and the subgroup spacing of the chimpanzees in this study was therefore compared to the dimensions of their previous housing. At the simplest level, subgroups could be expected to use either a similar amount of space or more space than their previous housing. On this basis, the subgroup formations were classified according to whether they were within or exceeded the original dimensions of their former enclosure (a 10 m × 10 m square; refer to Figure 1) with a 1 m edge effect, resulting in an 11 m × 11 m square, against which subgroups were compared. All classifications were based on the two-dimensional space occupied by the subgroups, such that individuals in elevated positions, such as trees, were still considered part of subgroups on the ground below them, provided they were in visual contact. If the subgroup fell within the 11 m × 11 m square it was labeled as a ‘small subgroup’, otherwise if a subgroup extended beyond the 11 m × 11 m square, it was labeled as a ‘large subgroup’.

For all subgroups, we recorded the sum of the inter-individual distances of chimpanzees on the periphery of the group, with an additional 1 m edge effect (referred to as the ‘subgroup polygon’). Other factors, such as proximity to the visiting public [54] may influence subgroup spacing patterns. Many animals also appear to have a ‘personal space’ which determines aspects of group spacing patterns [40,55,56] and chimpanzees may use inter-individual distances to determine group spacing. Thus, for all subgroups, the minimum distance to the public, the minimum inter-individual distance and maximum inter-individual distance were recorded. In addition, we recorded in which of the sections of the enclosure the subgroup occurred and the relative proportion of available space of that enclosure section (with a 1 m edge effect) the subgroup occupied.

Shade availability had a significant effect on the behaviour of this chimpanzee group [57] and thus may influence subgroup spacing. Thus, the weather conditions (sunny: <50% cloud cover; cloudy: 50–90% cloud cover; overcast: 90–100% cloud cover), the time of day when behaviours were sampled (in the morning, midday or afternoon sampling) and the maximum temperature for that day wasrecorded. The degree of available shade in the enclosure was recorded at the start of the observation session by visually estimating the percentage of the enclosure that was shaded at the start of the session and classifying the degree of shade according to a 5-point scale (1: 0–25% shade; 2: 25–50% shade; 3: 50–75% shade; 4: 75–99% shade; 5: overcast). Furthermore, to estimate the potential thermal experience of each subgroup, a value was assigned according to the following index:

$$I_{s}=\frac{S}{D+S}$$

where *S* is the number of individuals in the shade and *D* is the number of individuals in sunlight. Individuals were considered to be in shade if any part of their trunk was shaded. These measures of shade and sun utilization were then compared to the subgroup size categories for each time slot. Overcast days were excluded from this analysis so as to minimize bias toward shade utilization, resulting in the omission of four days from the final analyses.

#### 2.2.4. Social Influences on Subgroup Spacing

In order to determine whether social factors might be governing subgroup spacing, the individual composition of subgroups was assessed and recorded using photographs to identify individuals wherever possible to generate a record of subgroup composition. We also recorded which individuals were not part of the respective subgroups. All possible pair associations and non-associations were scored per subgroup, such that each subgroup composition was summarised as a number of pair-wise inter-individual associations. Thus, for every observation session a matrix was generated with the number of times that every possible pair combination of individuals occurred or did not occur within a subgroup.

### 2.3. Data Analysis

All analyses were conducted using Statistica 7 [58] unless otherwise stated. All tests were two-tailed and test significance was set at 0.05. We used generalized linear model (GLZ) analyses in which the response variable states were mutually exclusive (e.g., a subgroup either fits within a 11 m × 11 m space or did not; therefore, it was either a large subgroup, or a small one, but cannot be both simultaneously), and as such were coded as counts of the two states (e.g., small or large subgroups) of the variable in question. This resulted in a binomial presence/absence count for each variable. This binomial presence/absence structure was then used as the response variable in the GLZ analyses.

#### 2.3.1. Enclosure Space Use

Following calculation of the spread of participation index (SPI), space-use patterns were examined by comparing the observed and expected frequencies of zone use for the three time periods (morning, midday, afternoon). Because the perceived relative value of specific environmental features for the chimpanzees is unknown, expected frequencies were calculated based on the area of each zone, assuming homogenous space use. Zone size was calculated using the scale map and SimplePCI software [59]. For both observed and expected frequencies, the number of hits (number of times an individual was present in the zone) and misses (number of times an individual was not present in the zone) were calculated. In addition to the statistical analysis described below, zone bias was calculated, for all the data pooled and for individual time periods, by subtracting the expected hits from the observed hits and was plotted graphically.

Space-use records were analysed using a GLZ with a binomial error structure and logit link function. The time of day (morning, midday, afternoon), zone (A–H; Figure 1) and frequency type (observed, expected) were used as categorical predictors, while the binomial counts of hits and misses per zone wereused as the response variable. In order to directly address the aims of the study, only the appropriate second-order and third-order interaction effects were examined in detail. Significant differences within the second-order and third-order effects were identified through β-estimate coefficients and confidence intervals.

#### 2.3.2. Subgroup Spacing Null Model

To determine whether the number of small subgroups was random, a randomized null model using Monte Carlo sequences was compared to the number of observed small subgroups through a 1000 iteration randomization test. The outcome of the randomization test suggested that the observed subgroups formation was not random. In addition, a χ^2^ test was run to analyse whether the occurrence of small and large subgroups differed from chance.

#### 2.3.3. Behavioural Effects on Subgroup Spacing

The behaviour of individuals may influence the formation of subgroups of a particular size. For example, behaviours such as allo-grooming necessitate direct physical contact and close spacing and thus a small subgroup formation is more likely to occur when subgroup members engage in such activities. Alternatively, behaviours such as travelling are not likely to encourage tight spacing as this might hinder movement. Thus, in order to establish whether particular behaviours were driving the formation of small and large subgroups, we used a GLZ with a logit link function and binomial error structure. The behaviour (Table 1) was used as the categorical predictor and the counts of occurrences of that behaviour in small and large subgroups was coded as the binomial dependent (absent/present) response variable. β-estimate coefficients and confidence limits were used to assess specific differences between first order (behaviour) effects.

#### 2.3.4. Environmental Effects on Subgroup Spacing

The effects of environmental and spatial factors on subgroup spacing patterns were examined using a backward-stepwise GLZ with a logit link function and binomial error structure. The variables examined were assigned according to Table 2. β-estimate coefficients and confidence limits were used to assess specific differences between first-order (time of day, section of enclosure, subgroup type) and second-order (time of day × section of enclosure, section of enclosure × subgroup type, time of day × subgroup type) effects. Significant continuous predictors were correlated to subgroup size using a Spearman’s Rank Order Correlation.

#### 2.3.5. Social Effects on Subgroup Spacing

To assess social pair associations and non-associations which might influence subgroup spacing, a cellwise comparison using adjusted residuals was run to identify social associations in MatMan^TM^ [60]. Following this, five significant social pair associations (i.e., individuals that participated in subgroups more frequently than expected by chance alone; Daisy and Joyce; Daisy and Zoe; Daisy and Lilly; Zoe and Charles; Zoe and Joyce; hereafter referred to as ‘key associations’) and 14 social pair non-associations (i.e., individuals that participated together in subgroups less frequently than expected by chance alone; Thabuand Daisy; Thabuand Joyce; Thabuand Charles; Daisy and Yoda; Yoda and Joyce; Yoda and Charles; Yoda and Lilly; Yoda and Amber; Yoda and Zoe; Zoe and Lilly; Zoe and Amber; Lilly and Amber; Amber and Joyce; Amber and Charles) were identified. Based on the identified key associations, we investigated whether key associations predicted the formation of subgroups, regardless of size. This was done by performing a series of two-tailed χ^2^ tests to assess the number of times a pair was part of any-sized subgroup compared to; (i) the number of times they could have been part of a subgroup (it is possible for more than one subgroup to form simultaneously), and (ii) the times subgroups formed.

In order to determine whether key associations might predict the formation of small subgroups specifically, we then ran a series of two-tailed χ^2^ tests to examine the following relationship:

$$\frac{\mathrm{Key}\;\mathrm{Association}\;\left(\mathrm{Small}\;\mathrm{Subgroups}:\mathrm{Large}\;\mathrm{Subgroups}\right)}{\mathrm{All}\;\mathrm{subgroup}\;\mathrm{formations}\;\left(\mathrm{Small}\;\mathrm{Subgroups}:\mathrm{Large}\;\mathrm{Subgroups}\right)}$$

For example, if a total of 14 small subgroups and 3 large subgroups are recorded, while 6 of the small subgroups and 2 of the large subgroup formations involve key associations, a comparison of the 6:2 key association subgroups and the 14:3 total subgroups was then analysed using a χ^2^ test. This would suggest whether the proportion of small: large subgroups involving key associations differed to the overall proportion of small: large subgroups, regardless of which individuals participated.

## 3. Results

### 3.1. Space Use

**Figure 2:**
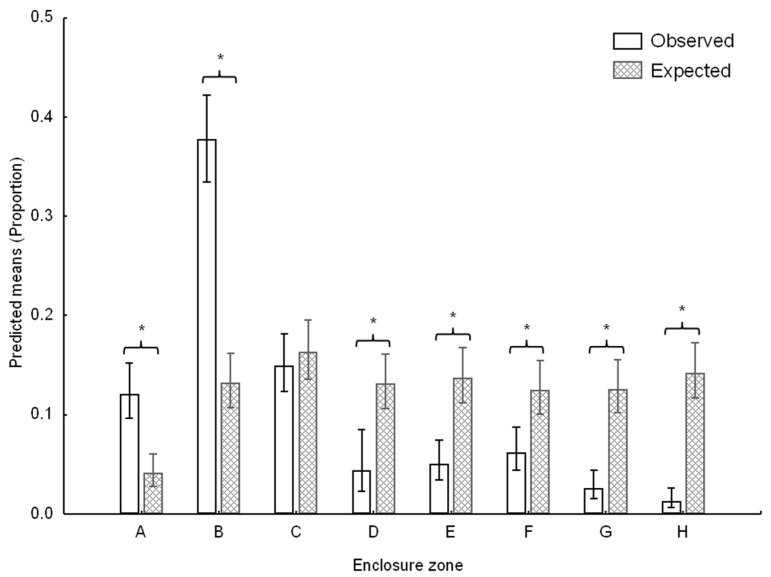
Observed and expected frequencies of zone use for eight zones in the chimpanzee enclosure at the Johannesburg Zoo Ape House. Bars denote predicted means proportions while whiskers denote confidence limits. Stars and brackets above bars denote significant (*p <* 0.05) differences between observed and expected zone use.

**Figure 3:**
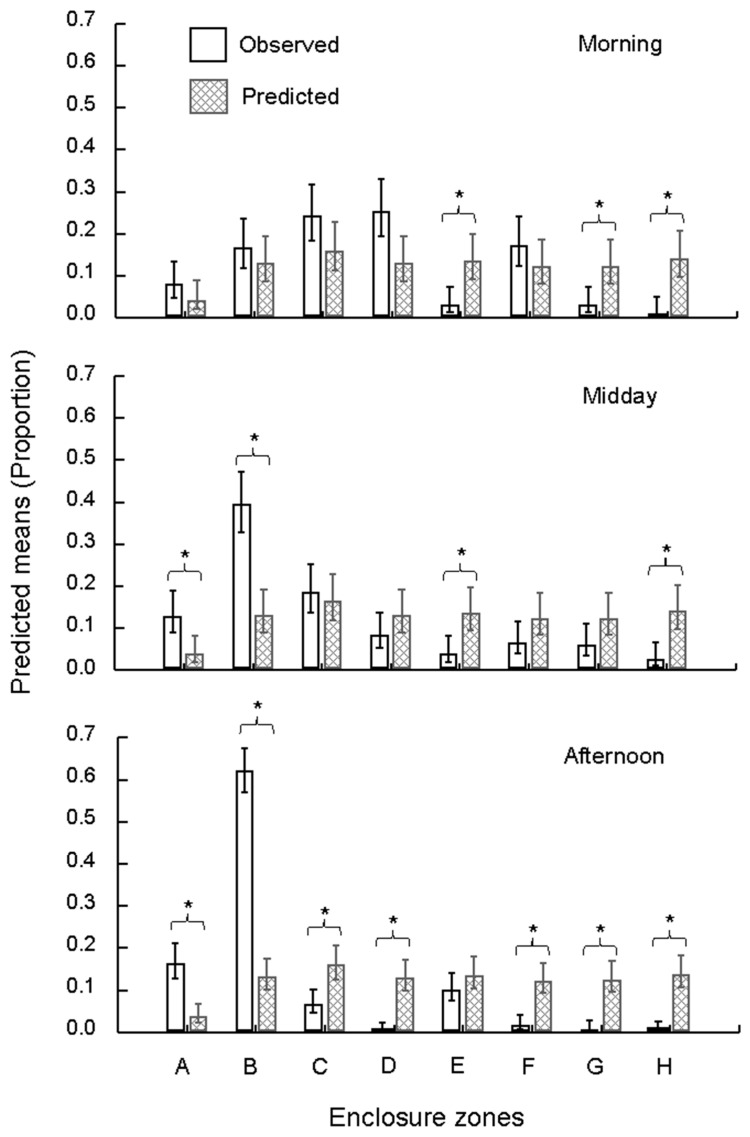
Zone-use patterns for the chimpanzees at the Johannesburg Zoo Ape House. Patterns of space use are represented for three time periods. Bars denote predicted means proportions for hits and misses in each zone while whiskers denote confidence intervals. Stars and brackets denote significant differences (*p* < 0.05) between observed and expected counts.

Time of day (Wald χ^2^_2_ = 15.13; *p <* 0.001), enclosure zone (Wald χ^2^_7_ = 230.05; *p <* 0.001) and frequency type (i.e., observed VS expected: Wald χ^2^_1_ = 38.97; *p <* 0.001) were all significant predictors of zone-use counts. In addition, the time of day × enclosure zone (Wald χ^2^_14_ = 112.88; *p <* 0.001), time of day × frequency type (Wald χ^2^_2_ = 15.11; *p <* 0.001) and enclosure zone × frequency type (Wald χ^2^_7_ = 221.23; *p* < 0.001; Figure 2) interaction effects were significant predictors of the zone-use patterns. Zones A and B were significantly overutilised while zones D–H were significantly underutilised in relation to the expected patterns of zone use based on the area of each zone (Figure 2). The time of day × enclosure zone × frequency type (Wald χ^2^_14_ = 111.6; *p <* 0.001; Figure 3) was also a significant predictor of the model outcomes with zones E, G and H significantly underutilised in the morning, zones E and H significantly underutilised around midday and zones C, D, F, G and H significantly underutilised in the afternoon. Zones A and B were significantly overutilised both at midday and in the afternoon (Figure 3).

### 3.2. Subgroup Formation

A total of 1285 subgroups were recorded with an average of 21.4 ± 4.6 subgroups recorded per observation session (60 min, 12 time slots) and 1.8 ± 0.7 subgroups recorded per time slot.The average number of individuals per subgroup was 3.2 ± 1.4 individuals. The results of the Monte Carlo sequence null model randomization test showed that the observed patterns of subgroup formation were not random (*p* < 0.0001). The resulting *p*-value is considered significant because it is not greater than the level of significance (α = 0.050) of the model [61], and thus the null hypothesis of no difference between the treatments (small, <11 m × 11 m, and large, >11 m × 11 m, subgroups) is rejected. In addition, significantly more small subgroups (1254 small subgroups) formed than large subgroups (31 large subgroups; χ^2^_1_ = 752.26; *p <* 0.0001).

#### 3.2.1. Behaviour

Behaviour was not a significant predictor of subgroup type (Wald χ^2^_7_ = 0.059; *p* = 1.000), indicating that behaviours that encouraged or required smaller inter-individual distances, such as allogrooming, were not likely to influence subgroup spacing.

#### 3.2.2. Environmental and Space Factors

Time of day, section of enclosure and the time of day × section of enclosure interaction were not good predictors of subgroup size (Table 2). None of the continuous predictor variables were significant predictors of subgroup size with the exception of subgroup polygon, which was weakly positively associated with increasing subgroup size (Table 2) and minimum distance to the public, which was weakly negatively associated with increasing subgroup size (Table 2).

#### 3.2.3. Social Influences on Subgroup Formation and Spacing

Five significant pair associations and 14 significant pair non-associations were identified (χ^2^_41_ = 1437.33; Table 3). For the five key associations, the proportion of total subgroups that formed was significantly different to the proportion of subgroups in which the pair participated (Daisy and Joyce χ^2^_1_ = 44.54, *p <* 0.001; Daisy and Zoe χ^2^_1_ = 39.54, *p <* 0.001; Daisy and Lilly χ^2^_1_ = 29.16, *p <* 0.001; Zoe and Charles χ^2^_1_ = 34.97, *p <* 0.001; Zoe and Joyce χ^2^_1_ = 31.84, *p <* 0.001). Thus, the frequency of participation in small subgroups by key associations did not match the frequency of small subgroup formations generally, indicating that key association pair participation was not a good predictor of subgroup formation.

With the exception of one pair (Zoe and Charles; χ^2^_1_ = 3.61, *p* = 0.057), the proportion of small to large subgroups involving key associations was significantly different from the overall small to large subgroup formations (Daisy and Joyce χ^2^_1_ = 5.53, *p* = 0.019; Daisy and Zoe χ^2^_1_ = 8.53, *p* = 0.004; Daisy and Lilly χ^2^_1_ = 25.7, *p <* 0.001; Zoe and Joyce χ^2^_1_ = 10.96, *p <* 0.001). This suggests that the proportion of small to large subgroups that involved Zoe: Charles was similar to the overall proportion of small to large subgroup formations.

## 4. Discussion

Our study sought to quantify the space use of a group of captive chimpanzees 5 years following the reconstruction of their outdoor enclosure, from a small (100 m^2^), barren enclosure to a large (2500 m^2^), naturalistic enclosure, at the Johannesburg Zoo. Firstly, the study described the space use of the chimpanzees in eight unequally-sized zones of the enclosure and compared the observations to expected frequencies of zone use based on zone size. Previous studies of chimpanzee space use have suggested that chimpanzees will not underuse enclosures [25] but, in agreement with other findings [39], we found that space use was not homogenous, with zones A and B being used more frequently than expected based on zone size. A pattern of temporal bias in space use emerged with all zones, apart from A and B, being underused during the midday and afternoon periods while morning space use was most evenly distributed.

An obvious explanation for the observed temporal patterns of space is the feeding regime of the chimpanzees. The scattering of food randomly throughout the enclosure appears to have successfully encouraged the chimpanzees to move throughout the available space. Scatter feeding is a relatively common, easy and effective environmental enrichment strategy employed for captive primates [62,63,64]. However, satiety and fooddepletion are pitfalls of feeding-based enrichment [65] and it appears that the effect of scatter feeding on the space use of the chimpanzees in our study lasts only as long as the food does, as evidenced by the more even space use in the morning when food is available and the more biased space use throughout the rest of the day. Thus the scatter feeding does appear to influence the space use of the chimpanzees but for a limited duration only.Nonetheless, the chimpanzees may have benefited from further scatter feeding throughout the day or through the baiting of the artificial termite mounds.

The occupancy of most zones was variable with zones being used at expected levels (morning: C, D, F; midday: C, D, F, G; afternoon: E) or levels lower than expected based on zone size (morning: E, G, H; midday: E, H; afternoon: C, D, F, G, H). Aside from the apparent preference for zones A and B (see below), there was no clear pattern in the use of these zones apart from the consistent avoidance of zone H. Zone H offered no vegetative cover, constant visual access to zoo visitors and only the un-baited artificial termite mound for climbing, suggesting little to attract the chimpanzees to this zone. Cover [44] and shade [57] are known to be important for captive chimpanzees and the presence of zoo visitors may have variable effects (see below). Zones C and D offered vegetative cover and climbing opportunities and, while our study did not consider three dimensional space use due to a relative lack of climbing opportunities, the presence of trees may offer apossible explanation for the patterns of use of these zones. These patterns support the idea that vegetative and visual cover are important for captive chimpanzees and should be considered in the captive management of primates [66]. The relative importance of climbing structures and vegetation, as well as the three dimensional space they provide, should be considered in future studies of space use. Furthermore, zoo managers should consider the use of enrichment to increase and homogenize space use in captive animals.

Further to the above, zones F, G and H also provided visual access to the adjacent orangutan enclosure. Previous studies have indicated that noise from neighbouring conspecifics influences chimpanzee behaviour [67,68] and auditory and olfactory contact with potential predators can influence behaviour and urinary corticosteroid levels in felids [69]. However, the orangutans never vocalized during our study and the chimpanzees appeared to ignore them. Moreover, habituation is also possible in such scenarios [70] and the two species had been neighbours for more than five years following the construction of the new enclosures, suggesting habituation to each other’s presence is likely. While visual contact with the orangutans may have influenced the chimpanzees’ use of these zones, the apparent lack of interest by the chimpanzees in the orangutans suggests that the neighbouring primates had little effect on the space use of the chimpanzees.Even so, it is still possible that the chimpanzees exhibit a learned avoidance of the orangutans.

The apparent preference of the chimpanzees for zones A and B is less straight forward. By contrast, zones A and B were used at expected levels in the morning or at levels higher than expected based on zone size in the midday and afternoon periods. Previous studies have reported that chimpanzees exhibit a preference for areas with doorways and corners [37,38], and zone B had two doorways: a night room entrance and the interconnecting doorway between the two enclosure sections. Zone E had a similar structure to zones A and B but had three doorways (two night room and one interconnecting), but was consistently underused, suggesting that perhaps the attraction to doorways and corners did not fully explain the relatively higher frequency of use of zones A and B in our study.

It is also possible that anticipation of their evening feed may have caused the chimpanzees to congregate in zones A and B, given that anticipatory behaviour prior to opportunities to feed and play occurs in other species [71,72,73]. We did not explicitly test for zone-specific behaviour patterns and thus we cannot say with certainty that anticipatory behaviour was evident. Anticipatory behaviour typically involves relatively brief periods of increased activity and locomotion [71,72,73,74], a behavioural pattern which might increase variation in zone use rather than limit the chimpanzees to a relatively small area of their enclosure. Further study would be required to investigate the role of anticipatory behaviour in space use patterns.

Captive apes may prefer areas with opportunities for human interaction [37], which they may experience as enriching [75], a view which is not universally held [76,77]. If visitor presence was enriching (as suggested by previous findings with this chimpanzee group [78]), the chimpanzees may have been attracted to zone A due to the large windows in the wall at that location which provided opportunity for close visual interactions with both zoo visitors and workers. However, were this the case, one would predict that zones D, F, G and H, zones with more extensive interactive and visual access to humans across the moats, would have been used more often than was observed. Furthermore, this does not explain the relative overuse of zone B. Alternatively, if visitor interactions were perceived as stressful, one would expect that neither A nor B would be overused as both zones provided open, constant visual access to zoo visitors at relatively close quarters and chimpanzees are known to avoid open areas [38]. Similarly, one might expect that the chimpanzees may have been attracted to zone B by keeper activity in the night rooms during cleaning. However, cleaning was typically brief and limited to the morning period and zone E would have offered equal if not more auditory cues of keeper activity, suggesting that it does not offer a good explanation for the preference for zones A and B throughout the day.

Together, the arguments above suggest that some other factors contributed to the observed pattern of space-use bias. While novelty may influence behaviour and space use in captive primates [11,46], it seems highly unlikely that neophobia is limited the space use of the chimpanzees several years after the enclosure change and they readily moved throughout the enclosure, suggesting that the space was not perceived as aversive. It is curious that the zones used most frequently coincided with the location of the original 10 m × 10 m enclosures (Figure 1). Following their initial release into the new enclosures, the current chimpanzee group was housed on the side of the enclosure abutting zones A-D and this may explain why the chimpanzees preferentially used A and B over E. Familiarity with the structural elements of the old housing incorporated into the new enclosures may have attracted the chimpanzees to this location. Common marmosets *Callithrix jucchus* and cotton-top tamarins *Saguinusoedipus* moved to open outdoor environments limited their space use to those areas around the entrances to their previous, indoor cage housing and the latter typically ventured no more than 3 m from the indoor cage entrance, an effect which apparently was due to a lack of cover in the environment [16]. The response of the chimpanzees appeared to be similar to these other primates but cannot be explained by lack of cover given that zones C–H provided far more cover than zones A and B.

It is also important to recognize that our study covered a short period over the austral autumn-winter seasons. Previous studies have reported seasonal effects on chimpanzee behaviour [11] and the chimpanzees in this study are known to use space according to their thermal needs [57]. Thus, while this study took place over a period of relative seasonal stability, there is the possibility that season may influence space use patterns and warrants further examination in future studies.

The second aspect of the space use of the chimpanzees which we investigated was the subgroup spacing of the chimpanzees. The chimpanzees appeared to consistently form tightly spaced, small subgroups (<11 m × 11 m) rather than large (>11 m × 11 m) subgroups in the outdoor enclosure. The formation of small subgroups was non-random, with significantly more small (97.5%) than large (2.5%) subgroups, which was not predicted by observed behaviour, time of day, enclosure section, maximum daily temperature, shade availability, the proportion of the total available space being used or the maximum and minimum inter-individual distances. However, two significant predictors of small subgroup formation were identified: subgroup polygon and minimum distance to the public.

While subgroup polygon and minimum distance to the public emerged as significant predictors of subgroup spacing patterns, neither appeared to explain the observed patterns of subgroup spacing. The relationship between subgroup polygon with subgroup size was weakly positive. However, logically, as subgroups form over a small area, they are more inclined to have a smaller polygon. Thus, the relationship between these factors does little to clarify the causality of small subgroup formation.

A weakly negative association emerged between subgroup size and minimum distance to the public, such that subgroups formed over a larger area in relatively closer proximity to the public. Interactions with zoo visitors are vital to further interests of zoos [79] but evidence from a variety of studies suggests that public interactions may be stressful for non-human primates [80]. Yet, chimpanzees will voluntarily interact with the public for extended periods [81] and readily exchange objects with humans [82], suggesting that these interactions are not necessarily as stressful for chimpanzees as for other captive primates. However, direct public interactions by the chimpanzees were limited to zones D, G and H and usually through begging behaviour, which was occasionally successful, and it appears that the formation of larger subgroups during such interactions could function to reduce inter-individual competition for food.Widely distributed resources are associated with less aggression [83] whereas restricted access to food can cause increased aggression [84].Thus the chimpanzees may increase their inter-individual spacing as a means of minimizing potential conflict. Spacing and territoriality in free-living chimpanzees appear to be driven by food dispersal and resource competition [85] with subgroups forming in response to dispersed [86] but not abundant [87] resources. Regardless, public interactions occurred infrequently (2.5% of all observed behaviour), suggesting that this behaviour is not a likely driver of subgroup spacing.

Local enhancement constitutes the most basic form of social behaviour by causing aggregations of individuals at a location [88], and it is possible that one or more individuals might have attracted other group members to form small subgroups through this social process.When social factors were examined, none of the social pair associations of chimpanzees were good predictors of subgroup formations and only one pair of individuals (Zoe and Charles) participated in small:large subgroup formations at a similar proportion as overall small:large subgroup formations. While at first this suggests that the presence of this pair might drive small subgroup spacing, the fact that no pairs were associated at levels above chance with overall patterns of subgroup formation precludes this possibility. In addition, the Zoe and Charles subgroups (small = 228: large = 11) occurred at similar proportions to overall small:large subgroup formations (small = 1 254: large = 31), but more small subgroups formed in the absence of this pair than when they were present. Primate biology predicts that sociality might determine spacing patterns since primate societies are maintained through complex dominance hierarchies [89] and primates generally ascribe great value to their social relationships [42]. In addition, animal spacing patterns tend to be governed by individual-specific rules regarding personal space [40] and a study of macaques *Macaca mulatta* found that social factors accounted for the spacing patterns of first- to second-nearest neighbours [24]. The chimpanzees in this study were all closely related and it is possible that kin relationships may have influenced the spacing patterns of the group. However, kin effects were not evident from our analyses.Thus it is curious that social factors did not explain the subgroup spacing of the chimpanzees.

Data on the spacing patterns of free-ranging chimpanzees are sparse but free-ranging chimpanzees typically have large inter-individual distances (Jane Goodall, Pers. Comm.). Studies of free-ranging chimpanzees have considered individuals to be in the same subgroup with inter-individual distances of between 35 m [52] and 100 m [53], considerably larger than the observed patterns in our captive study. When comparing the space use of individuals, Hedeen [90] reported limited space use in a captive-born gorilla when compared with the space use of group-mates captured from nature and captive chimpanzees travel shorter daily distances than their free-ranging conspecifics [91]. These data suggest that captivity alters space use in apes and that the pattern observed was likely a consequence of some aspect of captivity. Unfortunately, while many studies of the space use of chimpanzeesand other apes describe patterns of zone occupancy or preferences for specific features of their enclosures [11,37,38,39,45,90], no studies have quantified the inter-individual spacing patterns of captive chimpanzees. Thus it is difficult to ascertain whether the observed pattern is common to all chimpanzees or an outcome of the past experience of our study population specifically.

It seems unlikely to be coincidental that the chimpanzees showed a preference for the areas of the previous housing and formed subgroups which almost always conformed to the dimensions of the previous housing. One potential explanation is that the observed patterns of space use resemble a form of spatial learned helplessness. Learned helplessness is the inability of a subject to overcome a deferred controllable stressor following exposure to an uncontrollable stressor [92]. The learned helplessness hypothesis suggests that when the reaction of an individual to a stimulus fails to generate an effect, it learns that the resulting outcomes are independent of its actions [93,94] which then impedes learning that the response and outcomes are linked [95] when an influence over the outcomes is possible [96]. The emergence of learned helplessness appears to be contingent upon the initial stimulus being uncontrollable [94], regardless of whether the initial stimulus is benign, neutral or noxious [95,96,97,98].

Three criteria characterise learned helplessness: (1) a failure to react appropriately to a stimulus, (2) difficulty learning that the individual’s responses to future stimuli may influence the events, and (3) that these two effects arise only under conditions where the initial stimulus is uncontrollable and not when the stimulus is controllable [97,99]. With regard to the first criterion, the chimpanzees in our study displayed consistently tight subgroup spacing as well as a tendency to use the zones in the vicinity of the original enclosure, despite the availability of a large space, similar to the escape failures described for dogs [100] and rats [101]. This type of reaction suggests an inability to learn that the previous experience, limited available space and associated restrictions on space use and subgroup spacing in this case, no longer applied in the larger enclosure, fulfilling the second criterion. Controllability of the initial stimulus is crucial to the onset of learned helplessness [93,94,96,97,100,102,103] but it was not possible to generate a ‘controllable’ spatial change of this type for the chimpanzee group, and thus, the third criterion for learned helplessness cannot be explicitly confirmed for the chimpanzee group. However, the enclosure change was uncontrollable for the chimpanzees and thus, based on the existing evidence, the behaviour of the chimpanzees appeared to meet the criteria for learned helplessness (Martin Seligman, Pers. Comm.). Moreover, the spacing patterns described here in the chimpanzees mirror anecdotal descriptions of learned helplessness in pike (pike placed into a tank with guppies, but separated from the guppies by a glass barrier, failed to move through the full tank when the barrier was removed [104]) and fleas (fleas placed into a closed jar initially jump but soon stop, even once the lid is removed, having learned the physical limits on their locomotion due to the jar lid [105]). Similarly, such an effect can also be experimentally induced in common woodlice *Porcellio scaber* and African striped mice *Rhabdomys dilectus dilectus* [106] and experimental work on jumping spiders *Phidippus audax* demonstrated similar effects of rearing enclosure size on various aspects of spider spatial behaviour [107].

Curiously, the space-use bias and tight spacing patterns of the chimpanzees occurred despite the fact that the larger enclosure was not necessarily a deferred noxious stimulus. It is possible that the large space of the new enclosure is perceived as stressful, but the chimpanzees readily travelled independently of one another throughout the enclosure and the new enclosure had persistent ameliorating effects on various stress-related behaviours immediately after, and 10 weeks following, the release into the new enclosures [41], suggesting that this is not the case. Instead, we would argue that the experience of the chimpanzees in the previous restricted housing influenced their perception of the space available to them, resulting in a spatio-perceptual deficit, manifesting as a self-imposed ‘invisible cage’ which limited their space use.

The idea that past experience and learning may have lasting effects on future behaviour, a long-term effect which has been demonstrated in chimpanzees [108], is not novel, and is a mechanism for the manifestation of many stereotypic behaviours in captive animals [109]. We are not suggesting that learned helplessness is present in all captive populations or that it definitively explains the patterns we report in our study; rather we are suggesting that, given that alternative explanations appear not to explain the observed patternsadequately, learned helplessness provides a potential explanation for these patterns of space use and subgroup spacing which warrants further study. To the best of our knowledge, ours would constitute the first formal quantification of this effect in a spatial context. The role of past experience on later space use requires further investigation under controlled experimental conditions but, if our proposed learned helplessness interpretation has merit, this raises several important questions. In the context of our study specifically, how persistent is the “invisible cage effect” likely to be? Might cultural transmission effects perpetuate the observed pattern of self-imposed spatial restriction? Our study suggests that the effect persists in the long-term but further work is needed to determine the extent and mechanism of this. It also raises important questions around animal husbandry practice and enclosure design. Do animals housed in small enclosures necessarily gain welfare benefits from large enclosures per se, or is simply enriching a small enclosure sufficient? Visitor and animal caregiver perceptions aside, is it necessary for institutions to invest resources into the design and construction of large naturalistic enclosures if animals are unlikely to use the entire space? Controllability and choice is paramount to good animal welfare and one of the primary aims of enrichment of captive environments is to provide this [110]. We argue that even if animals do not use the entire available space or all available enrichment, the choice for a captive animal to use the space/enrichment or not affords the animal valuable control in an environment which is otherwise largely uncontrollable [111].

Chimpanzees are endangered [112] and, given the threats facing free-living populations of the species [113,114], their conservation through the maintenance of captive populations is paramount. While a learned helplessness effect may only occur in individuals which experience restrictive environments, what might the impact of learned helplessness be when captive individuals are reintroduced into nature?It is not difficult to imagine scenarios where natural behaviour might be compromised by learned helplessness effects. For example, long-term spatial memory is important for free-living chimpanzees to locate fruiting trees [115] and chimpanzees actively patrol and defend large home ranges in nature [116] but if the movements and spacing of individuals are limited by past spatial conditions, how might this impact on processes such as foraging or home range defence? Evidence already exists for these effects on free-ranging animals; African elephants *Loxodonta africana* exhibited a similar self-restricted space use following the removal of boundary fences in Phinda Private Game Reserve, South Africa, which persisted for at least a year [117]. This report, coupled with the findings of D’Egidio [106] and Carducci and Jakob [107] and the anecdotal reports of Beasor [104] and Ziglar [105], suggests that the effects of previous spatial restriction on space use are also not limited to chimpanzees and may have important implications for species ecology and conservation more broadly.

## 5. Conclusions

In conclusion, the chimpanzees at the Johannesburg Zoo limited their space use in their large, naturalistic, outdoor enclosure to those areas making up their former housing and chimpanzee subgroups conformed to the dimensions of the previous housing condition. This pattern of space use was not explained by several candidate predictors and appears to resemble a form of spatial learned helplessness. Our study is the first to describe such a pattern of space use and, because no studies have quantified the inter-individual spacing patterns of other captive chimpanzees, it is difficult to know whether the observed pattern is common to other captive populations or is an outcome of the past experience of our study group specifically; hence further research is warranted. Our study has identified many avenues for future research into the effects of spatial restriction on animals which may have important implications for captive animal welfare and conservation, particularly when animals are transferred to larger cages for enrichment or relocated into nature, as occurs in many rehabilitation and re-release programmes. Future studies should also focus on expanding the existing understanding of space use in captivity, those factors which influence it and how it can be manipulated to enhance the welfare of captive animals.

## Figures and Tables

**Table 1:** Definitions of behaviours sampled in the chimpanzee group at the Johannesburg Zoo.

Behaviour	Definition
Locomotion	Movement from one location to another, not involving searching for food. Included walking, running and climbing
Foraging	Activity related to the searching for, manipulation or consumption of food or drink
Socio-negative	Chasing aggressively (characterised by sneering, open and closed grins and compressed lip faces. Usually associated with screams, barks and “wraaa” calls [48]) or overt fighting. Included aggressive gesturing or signalling
Socio-positive	Affiliative behaviours such as social grooming and embracing directed at other chimpanzees
Play	Social play (wrestling, playful biting and playful chasing characterised by a relaxed face, possibly with a drooping lower lip, or a full play face. Usually silent but may include soft grunts or hoots [48]), object play (play directed at or involving an inanimate object) and locomotor play (solitary active play. Included running, rolling, swinging or somersaulting)
Inactivity	Resting, either standing or sitting down, or sleeping
Abnormal	Coprophagia/urophagia, self-mutilation, faeces throwing and hair plucking. Other behaviours were scored as abnormal based on the context in which they occurred and whether they occurred repetitively (>3 times in succession [49]). These included nipple pulling, abnormal gait and posturing and chronic masturbation
Public Interaction	Attempts by the chimpanzees to engage with the public through the viewing windows or fences
Hidden	Chimpanzees were obscured from view or behaviour was not identifiable according to the other categories listed

**Table 2:** The output of a generalised linear model analysis (Wald χ^2^), showing the effects of predictors listed for the assessment of subgroup size for chimpanzees at the Johannesburg Zoo. Variables and test statistics in bold indicate significant predictors of subgroup type. For significant continuous predictors, the Spearman’s rank order correlation (ρ) is shown.

Parameters	Wald χ^2^ Statistics	Spearman’s rho (ρ)
Time of day	χ^2^_2_ = 0.128; *p* = 0.938	
Section of enclosure	χ^2^_1_ = 0.01; *p* = 0.997	
Time of day × Section of enclosure	χ^2^_2_ = 0.931; *p* = 0.628	
Weather conditions at start of session	χ^2^_1_ = 3.240; *p* = 0.072	
Maximum daily temperature	χ^2^_1_ = 0.993; *p* = 0.319	
Percentage available shade in enclosure at start of session	χ^2^_1_ = 0.604; *p* = 0.437	
Shade index	χ^2^_1_ = 0.541; *p* = 0.462	
**Subgroup polygon (with 1 m edge effect)**	**χ^2^_1_ = 3.963; *p* = 0.047**	**R = 0.218; *p* < 0.05**
**Minimum distance to the public**	**χ^2^_1_ = 5.114; *p* = 0.024**	**R = −0.114; *p* < 0.05**
Maximum inter-individual distance	χ^2^_1_ = 1.137; *p* = 0.286	
Minimum inter-individual distance	χ^2^_1_ = 0.040; *p* = 0.841	
Proportion of enclosure section area used	χ^2^_1_ = 1.232; *p* = 0.267	

**Table 3:** Z-statistics for a χ^2^ using adjusted residuals used to examine associations between specific pairs of chimpanzees at the Johannesburg Zoo. Bold Z values within grey cells denote significant interactions and superscripts denote level of significance (^1^ *p* < 0.05; ^2^ *p* < 0.01; ^3^ *p* < 0.001).

		Non-Associations (Pairs That Formed Less Frequently than Expected by Chance)							
		Daisy	Thabu	Joyce	Charles	Yoda	Amber	Lilly	Zoe
**Associations (Pairs that formed more frequently than expected by chance)**	**Daisy**	-	**14.41 ^3^**	−1.29	0.10	**2.94 ^2^**	−0.48	−3.51	−8.61
	**Thabu**	−9.85	-	**11.07 ^3^**	**9.42 ^3^**	−0.76	−2.54	−2.60	−3.49
	**Joyce**	**4.15 ^3^**	−9.46	-	1.98	**4.90 ^3^**	**2.55 ^1^**	−0.90	−3.76
	**Charles**	1.55	−8.57	−1.10	-	**6.43 ^3^**	**3.34 ^2^**	1.35	−4.24
	**Yoda**	−4.63	0.47	−6.83	−8.28	-	**7.86 ^3^**	**3.15 ^2^**	**5.11 ^3^**
	**Amber**	−1.33	1.69	−5.08	−5.61	−8.47	-	**7.79 ^3^**	**8.55 ^3^**
	**Lilly**	**2.15 ^2^**	0.18	−1.24	−3.90	−2.79	−6.02	-	**10.70 ^3^**
	**Zoe**	**10.75 ^3^**	1.51	**3.24 ^2^**	**4.63 ^3^**	−5.23	−6.77	−4.86	

## Data Availability

The data used for this study can be made available by the authors on reasonable request.
